# Presence, function, and regulation of IL‐17F‐expressing human CD4^+^ T cells

**DOI:** 10.1002/eji.201948138

**Published:** 2020-01-16

**Authors:** Lachrissa A. Burns, Ash Maroof, Diane Marshall, Kathryn J. A. Steel, Sylvine Lalnunhlimi, Suzanne Cole, Anca Catrina, Bruce Kirkham, Leonie S. Taams

**Affiliations:** ^1^ Centre for Inflammation Biology and Cancer Immunology Department Inflammation Biology School of Immunology & Microbial Sciences King's College London London UK; ^2^ UCB Pharma Slough UK; ^3^ Department of Medicine Karolinska University Hospital Stockholm Sweden; ^4^ Dept Rheumatology Guy's and St Thomas’ NHS Foundation Trust London UK

**Keywords:** interleukin‐17, Th17, rheumatoid arthritis, psoriatic arthritis

## Abstract

The pro‐inflammatory cytokine IL‐17A has been implicated in the immunopathology of inflammatory arthritis. IL‐17F bears 50% homology to IL‐17A and has recently been suggested to play a role in inflammation. We investigated the induction and cytokine profile of IL‐17F^+^ CD4^+^ T cells, and how IL‐17F may contribute to inflammation. Upon culture of healthy donor CD4^+^ T cells with IL‐1β, IL‐23, anti‐CD3, and anti‐CD28 mAb, both IL‐17A and IL‐17F‐expressing cells were detected. In comparison to IL‐17A^+^IL‐17F^−^ CD4^+^ T cells, IL‐17F^+^IL‐17A^−^ and IL‐17A^+^IL‐17F^+^ CD4^+^ T cells contained lower proportions of IL‐10‐expressing and GM‐CSF‐expressing cells and higher proportions of IFN‐γ‐expressing cells. Titration of anti‐CD28 mAb revealed that strong co‐stimulation increased IL‐17F^+^IL‐17A^−^ and IL‐17A^+^IL‐17F^+^ CD4^+^ T cell frequencies, whereas IL‐17A^+^IL‐17F^−^ CD4^+^ T cell frequencies decreased. This was partly mediated via an IL‐2‐dependent mechanism. Addition of IL‐17A, IL‐17F, and TNF‐α to synovial fibroblasts from patients with inflammatory arthritis resulted in significant production of IL‐6 and IL‐8, which was reduced to a larger extent by combined blockade of IL‐17A and IL‐17F than blockade of IL‐17A alone. Our data indicate that IL‐17A and IL‐17F are differentially regulated upon T cell co‐stimulation, and that dual blockade of IL‐17A and IL‐17F reduces inflammation more effectively than IL‐17A blockade alone.

## Graphical abstract

High‐strength T cell stimulation enhances the frequency of IL‐17F^+^ CD4^+^ T cells in part via an IL‐2 dependent manner. Results demonstrate IL‐17A^+^ and IL‐17F^+^ CD4^+^ T cell populations display different cytokine profiles. Combined blockade of IL‐17A and IL‐17F is more effective at reducing inflammation than blockade of IL‐17A alone.

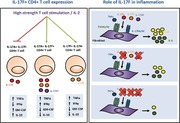

## Introduction

The IL‐17 family consists of IL‐17A (often referred to as IL‐17), IL‐17B, IL‐17C, IL‐17D, IL‐17E, and IL‐17F. IL‐17A is the best characterized IL‐17 family member that displays pro‐inflammatory functions and is implicated in the immunopathology of inflammatory arthritis including psoriatic arthritis (PsA), axial spondylitis (AxSpA), and to a lesser extent, rheumatoid arthritis (RA). IL‐17F shares a 50% homology to IL‐17A, however the induction, function, and presence of IL‐17F in health and inflammatory arthritis is less clear.

A well‐studied cellular source of IL‐17A and IL‐17F are CD4^+^ Th17 cells. In humans, TGF‐β, IL‐6, IL‐1β, or IL‐21 are required for Th17 differentiation, while IL‐23 is important for lineage maintenance and survival 1, 2. Previous data from our laboratory demonstrated that the frequency of IL‐17A^+^ CD4^+^ T cells is significantly increased when human memory CD4^+^ T cells are stimulated through the TCR in the presence of in vitro or in vivo activated monocytes 3, 4, 5. Reports have also demonstrated that low‐strength T cell activation favors the induction of human and mouse IL‐17A^+^ CD4^+^ T cells 6, 7. Relatively little is known regarding the factors that drive the induction of IL‐17F^+^ CD4^+^ T cells. However, it has been shown that similar to IL‐17A, human IL‐17F^+^ CD4^+^ T cells are enhanced in the presence of IL‐1β and IL‐23 1.

Reports have demonstrated Th17 cells are a heterogeneous population, including "non‐pathogenic" and "pathogenic" subtypes 8. In mice, TGF‐β and IL‐6 promote the generation of non‐pathogenic Th17 cells 8, whereas exposure to IL‐23 resulted in the conversion of nonpathogenic Th17 cells to pathogenic Th17 cells 9. Pathogenic Th17 cells are associated with a lack of IL‐10 production and enhanced GM‐CSF production 10. A recent mouse study showed that TGF‐β induced higher frequencies of IL‐17F^+^ CD4^+^ T cells 11. However, the cytokine profile associated with IL‐17F^+^ CD4^+^ T cells remains to be concluded.

IL‐17A and IL‐17F exert similar pro‐inflammatory effects, however, IL‐17A is more potent than IL‐17F 12. IL‐17A and IL‐17F can induce secretion of pro‐inflammatory mediators including IL‐6, IL‐8, and CXCL1 from fibroblasts and epithelial cells 12, 13. Both IL‐17A 14, 15, 16, 17 and IL‐17F 14, 16, 17 can amplify their inflammatory potential by synergizing with other cytokines including TNF‐α. In both the mouse collagen‐induced arthritis (CIA) and EAE model, blockade of IL‐17F did not exhibit an effect, whereas blockade of IL‐17A reduced inflammation and disease severity 12, 18. Dual IL‐17A and IL‐17F blockade in CIA had little additional effect compared to blockade of IL‐17A alone 18. However, in the experimental colitis mouse model neutralization of both IL‐17A and IL‐17F ameliorated colitis, whereas neutralization of IL‐17A or IL‐17F alone had limited effect 19. Of relevance, a recent report using human in vitro models suggest that dual blockade of IL‐17A and IL‐17F in Th17 supernatants was more effective at reducing the secretion of pro‐inflammatory mediators from target cells than blockade of IL‐17A alone 20. Finally, early clinical studies with bimekizumab, a dual IL‐17A and IL‐17F neutralizing antibody, have shown rapid and profound clinical responses in psoriasis 21 and PsA 20 patients suggesting an unappreciated role for IL‐17F in addition to IL‐17A in promoting inflammation.

While IL‐17A^+^ CD4^+^ T cells have been implicated in the immunopathology of inflammatory arthritis 22, 23, 24, robust evidence confirming the presence of IL‐17F^+^ CD4^+^ T cells or IL‐17F protein is lacking. A limited number of studies have identified the expression of IL‐17F in RA and PsA synovial tissue when compared with osteoarthritis (OA) samples via immunohistochemistry analysis 14, 25. In addition, a recent study showed detectable levels of *IL17F* mRNA in six out of 14 PsA synovial tissue samples 20. A different study, however, reported that while IL‐17A protein was detected in the supernatant of stimulated RA synovial fluid mononuclear cells, no IL‐17F protein was detectable 18.

Together these findings signify the need for a better understanding of the presence, function, and regulation of IL‐17F. Here, we sought to investigate what drives the induction of IL‐17F expression in CD4^+^ T cells, the cytokine profile of IL‐17F^+^ CD4^+^ T cells, how IL‐17F may contribute to inflammation, and the presence of IL‐17F and IL‐17F^+^ CD4^+^ T cells in inflammatory arthritis.

## Results

### Induction of IL‐17F expression in human CD4^+^ T cells

We first sought to investigate the presence of IL‐17F expressing CD4^+^ T cells in human blood. Healthy control CD4^+^ T cells from human blood were stimulated ex vivo for 3 h with PMA/ionomycin in the presence of Golgi‐Stop. IL‐17A^+^ CD4^+^ T cells were detected in all seven donors (ranging from 0.2 to 1.9%, Supporting Information Fig. 1). In contrast, only low frequencies of IL‐17F^+^ CD4^+^ T cells were detected (range 0.01–0.33%).

**Figure 1:**
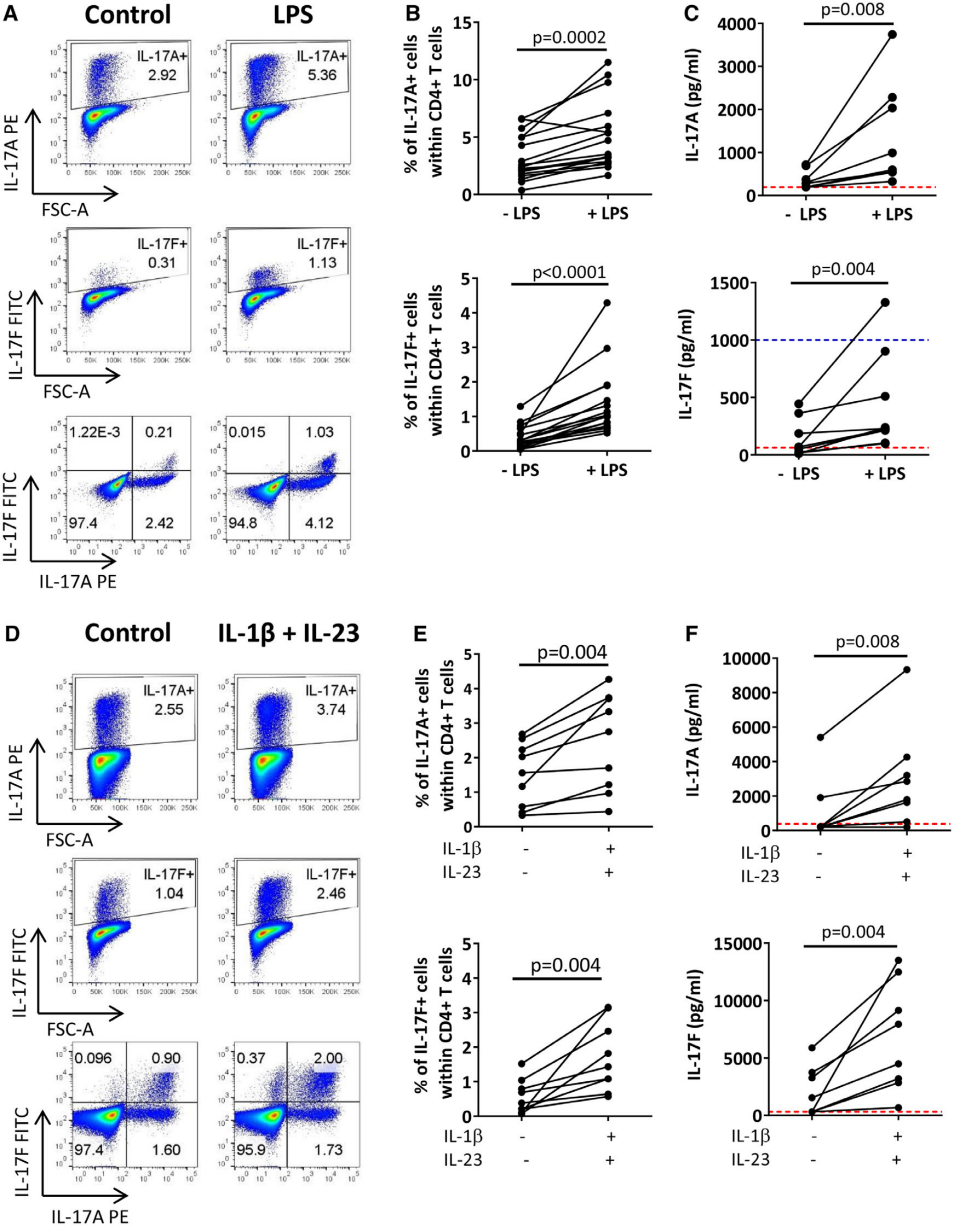
LPS‐activated monocytes and IL‐1β and IL‐23 enhance the frequencies of IL‐17A^+^ and IL‐17F^+^ CD4^+^ T cells. (A–C) Bulk CD4^+^ T cells (0.5 × 10^6^) were co‐cultured 1:1 with autologous monocytes and stimulated with anti‐CD3 mAb (100 ng/mL) in the absence (control) or presence (LPS) of LPS (100 ng/mL). After 3 days cells were re‐stimulated and assessed for intracellular cytokine expression by flow cytometry. (A) Representative dot plots and (B) cumulative data show frequencies of IL‐17A^+^ and IL‐17F^+^ cells within the total CD4^+^ T cell population (12 independent experiments using *n* = 17 donors in total). (C) Levels of IL‐17A and IL‐17F detected in culture supernatant (collected prior re‐stimulation) via ELISA (data from seven independent experiments using *n* = 9 donors in total). (D–F) Bulk CD4^+^ T cells were cultured with plate‐bound anti‐CD3 mAb (100 ng/mL) and anti‐CD28 mAb in the absence (control) or presence of IL‐1β (10 ng/mL) and IL‐23 (20 ng/mL). After 3 days cells were re‐stimulated and assessed for intracellular cytokine expression by flow cytometry. (D) Representative dot plots and (E) cumulative data show frequencies of IL‐17A (*n* = 9) and IL‐17F (*n* = 9) expressing cells within the total CD4^+^ T cell population (six independent experiments using *n* = 9 donors in total). (F) Cumulative ELISA data for IL‐17A and IL‐17F (data from five independent experiments using *n* = 8 donors in total). Dashed red and blue lines indicate lower and upper ELISA detection limits, respectively. Each connecting line represents a different donor. Data were analyzed using Wilcoxon matched‐pairs signed rank test.

To examine factors that could induce IL‐17F^+^ CD4^+^ T cells and IL‐17F secretion in vitro, we expanded on our previously published work, which assessed the effect of LPS‐activated monocytes on IL‐17A induction 3, 4, 5. CD4^+^ T cells derived from healthy human blood were co‐cultured with autologous CD14^+^ monocytes and stimulated with soluble anti‐CD3 mAb in the absence or presence of LPS for 3 days. Supernatants were collected for analysis of IL‐17A and IL‐17F protein via ELISA, and the remaining cells re‐stimulated with PMA/ionomycin and analyzed by flow cytometry. A representative gating strategy and fluorescence minus control (FM) plots are shown in Supporting Information Fig. 2. In concordance with our previous data, addition of LPS to T cell/monocyte co‐cultures led to a statistically significant increase in the frequency of IL‐17A^+^ CD4^+^ T cells (1.6‐fold, *P* = 0.0002) as well as in IL‐17A secretion (on average 368 vs. 1389 pg/mL; 3.2‐fold, *P* = 0.008). LPS stimulation also resulted in a 3.4‐fold increase in IL‐17F^+^ CD4^+^ T cell frequencies (*P* < 0.0001) and a 1.8‐fold increase in IL‐17F secretion (*P* = 0.004; on average 137 vs. 428 pg/mL; Fig. 1A–C). In this T cell/monocyte co‐culture system, two distinct IL‐17 populations were observed: IL‐17A^+^IL‐17F^−^ single‐positive and IL‐17A^+^IL‐17F^+^ double‐positive CD4^+^ T cells (Fig. 1A).

To examine the effect of LPS addition on other cytokines, co‐culture supernatants were analyzed by Luminex. Significant increases in IL‐1β, IL‐6, and IFN‐γ were observed in LPS‐treated conditions (Supporting Information Fig. 3). IL‐23 was also detected at elevated levels in five of nine experiments. To test whether IL‐1β and IL‐23 can drive IL‐17F expression in CD4^+^ T cells, bulk CD4^+^ T cells were cultured for 3 days with plate‐bound anti‐CD3 mAb, soluble anti‐CD28 mAb (replacing the co‐stimulation signal provided by CD14^+^ monocytes) in the absence or presence of IL‐1β and IL‐23. Addition of IL‐1β and IL‐23 led to a significant increase in the frequency of CD4^+^ T cells expressing IL‐17A (1.7‐fold, *P* = 0.004) and/or IL‐17F (3.7‐fold, *P* = 0.004; Fig. 1D and E), as well as significant increases in IL‐17A (on average 1068 vs. 2972 pg/mL; 2.8‐fold, *P* = 0.008) and IL‐17F protein levels (on average 1962 vs. 6788 pg/mL; 3.5‐fold, *P* = 0.008; Fig. 1F). In this T cell culture system, three distinct IL‐17 populations were observed: IL‐17A^+^IL‐17F^−^ single‐positive, IL‐17A^+^IL‐17F^+^ double‐positive, and IL‐17F^+^IL‐17A^−^ single positive CD4^+^ T cells (Fig. 1D).

### CD28 co‐stimulation and high‐dose anti‐CD3 mAb enhance induction of IL‐17F^+^ CD4^+^ T cells

**Figure 2:**
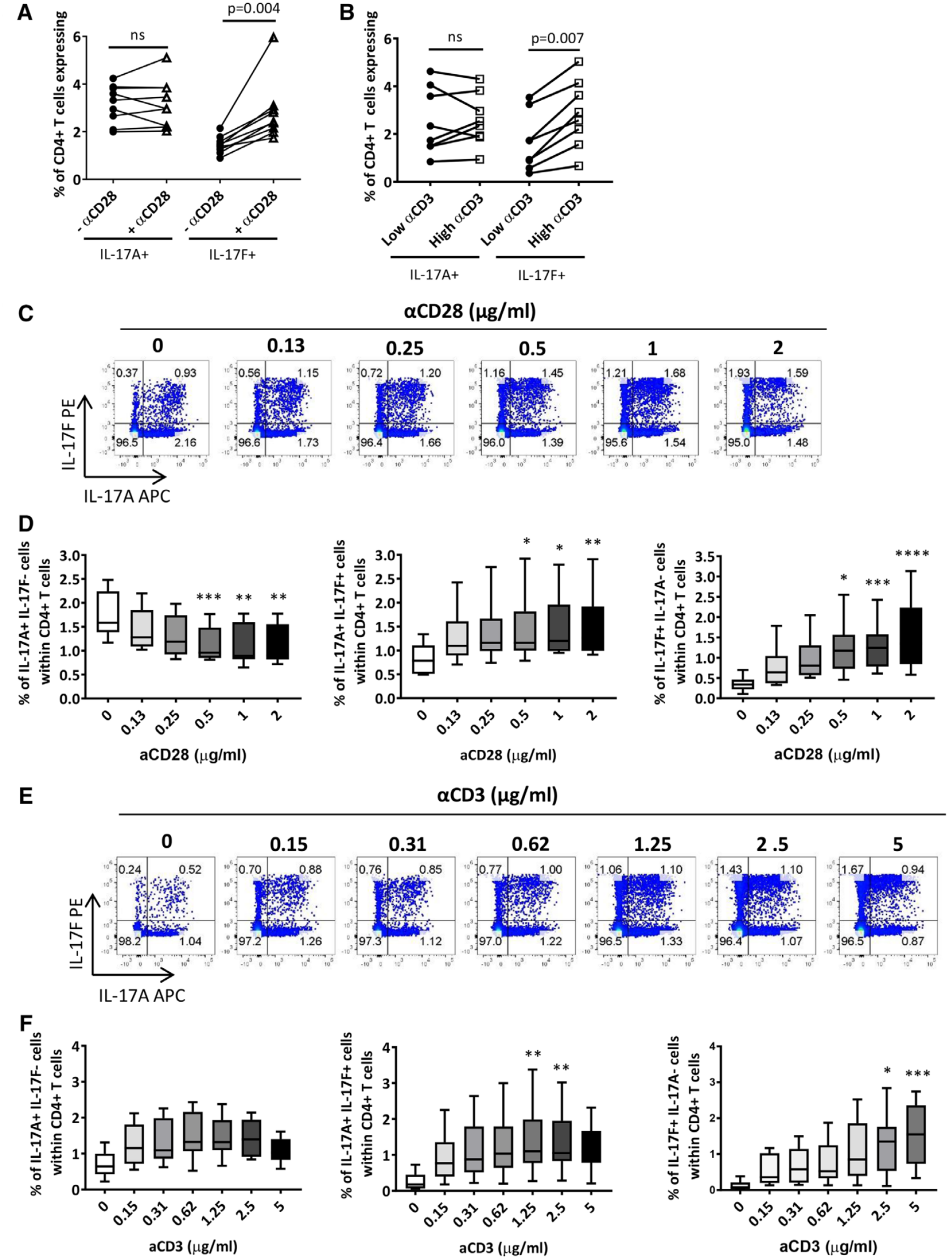
CD28 co‐stimulation and high‐dose anti‐CD3 mAb enhance induction of IL‐17F^+^ CD4^+^ T cells. (A) Bulk CD4^+^ T cells were cultured with anti‐CD3 mAb, IL‐23, and IL‐1β in the absence or presence of anti‐CD28 mAb (1 μg/mL). After 3 days, cells were re‐stimulated and assessed for IL‐17A and IL‐17F cytokine expression via flow cytometry. Results show the cumulative data for IL‐17A^+^ and IL‐17F^+^ CD4^+^ T cells (four independent experiments using *n* = 9 donors in total) analyzed using a Wilcoxon‐matched pairs test. (B) Bulk CD4^+^ T cells were cultured with anti‐CD28 mAb, IL‐23, and IL‐1β in the presence of low (0.15 μg/mL) or high (5 μg/mL) dose anti‐CD3 mAb for 3 days and then re‐stimulated. Results show the cumulative flow cytometric data for IL‐17A^+^ and IL‐17F^+^ CD4^+^ T cells (three independent experiments using *n* = 8 donors in total) analyzed using a Wilcoxon‐matched pairs test. Bulk CD4^+^ T cells were cultured with (C and D) anti‐CD3 mAb, IL‐23, and IL‐1β in the presence of increasing doses of anti‐CD28 mAb (0–2 μg/ml) or (E and F) anti‐CD28 mAb, IL‐23, and IL‐1β in the presence of increasing doses of plate‐bound anti‐CD3 mAb (0–5 μg/mL). After 3 days, cells were re‐stimulated and assessed for IL‐17A and IL‐17F cytokine expression. (C and E) Representative dot plots show the percentages of IL‐17A^+^IL‐17F^−^, IL‐17A^+^IL‐17F^+^, and IL‐17F^+^IL‐17A^−^ cells within CD4^+^ T cells and (D and F) box whisker plots represent cumulative data from (D) three independent experiments using *n* = 6 donors in total and (F) three independent experiments using *n* = 8 donors in total. Data are shown as the mean ± SEM. Statistical analysis was performed using Friedman test with comparison to control (0 μg/mL for anti‐CD28 mAb and 0.15 μg/mL for anti‐CD3 by Dunn's Multiple Comparisons test. *P < 0.05, **P < 0.01, ***P < 0.001, ****P < 0.0001.

To investigate the contribution of CD28 co‐stimulation to the induction of IL‐17F^+^ CD4^+^ T cells, healthy peripheral blood bulk CD4^+^ T cells were cultured with plate‐bound anti‐CD3 mAb, IL‐1β, and IL‐23 in the absence or presence of 1 μg/mL anti‐CD28 mAb for 3 days. Addition of anti‐CD28 mAb had no effect on the frequency of total IL‐17A^+^ CD4^+^ T cells, while significantly increasing the frequency of total IL‐17F^+^ CD4^+^ T cells (Fig. 2A). To assess the effect of anti‐CD3 mAb stimulation, bulk CD4^+^ T cells were cultured with IL‐1β, IL‐23, and anti‐CD28 mAb with either low (0.15 μg/mL) or high (5 μg/mL) doses of plate‐bound anti‐CD3 mAb. While no effect in percentage of IL‐17A^+^ CD4^+^ T cells with low versus high doses of anti‐CD3 mAb was observed, high dose anti‐CD3 mAb significantly increased the frequency of IL‐17F^+^ CD4^+^ T cells (Fig. 2B).

We extended these observations by titrating anti‐CD28 and anti‐CD3 mAbs into CD4^+^ T cell cultures, in presence of IL‐1β and IL‐23. Titration of anti‐CD28 mAb led to a dose‐dependent decrease in the percentage of IL‐17A^+^IL‐17F^−^ CD4^+^ T cells, while increasing IL‐17A^+^IL‐17F^+^ and IL‐17F^+^IL‐17A^−^ CD4^+^ T cells (Fig. 2C and D). Titration of anti‐CD3 mAb also increased the frequency of IL‐17A^+^IL‐17F^+^ and IL‐17F^+^IL‐17A^−^ CD4^+^ T cells in a dose‐dependent manner (Fig. 2E and F). When examining cytokine secretion in cell culture supernatants, titration of anti‐CD28 mAb led to a dose‐dependent increase in both IL‐17A and IL‐17F protein secretion (Supporting Information Fig. 4A). Similar results were observed with titration of anti‐CD3 mAb (Supporting Information Fig. 4B). Higher levels of IL‐17F versus IL‐17A were detected, although this should be interpreted with caution as different ELISA antibody affinities make it difficult to draw comparisons between levels of different cytokines.

The increase in IL‐17A secretion was unexpected as our flow cytometry data suggested IL‐17A expression remained unchanged with higher doses of anti‐CD28 mAb or anti‐CD3 mAb. This result could be due to the kinetics of the assay and reflect accumulation of IL‐17A secreted in the early stages of CD4^+^ T cell activation. To investigate the kinetics of IL‐17A and IL‐17F expression from CD4^+^ T cells, healthy control CD4^+^ T cells were cultured with plate‐bound anti‐CD3 and soluble anti‐CD28 for various time points (0–80 h) followed by culture for 3 h in the presence of brefeldin. As shown in Supporting Information Fig. 5, IL‐17A appears to peak at the early stages of CD4^+^ T cell activation, while IL‐17F expression shows a more gradual increase, with high expression observed at the later stages of CD4^+^ T cell activation.

### CD28‐driven induction of IL‐17F^+^ CD4^+^ T cells is mediated in part by IL‐2

**Figure 3:**
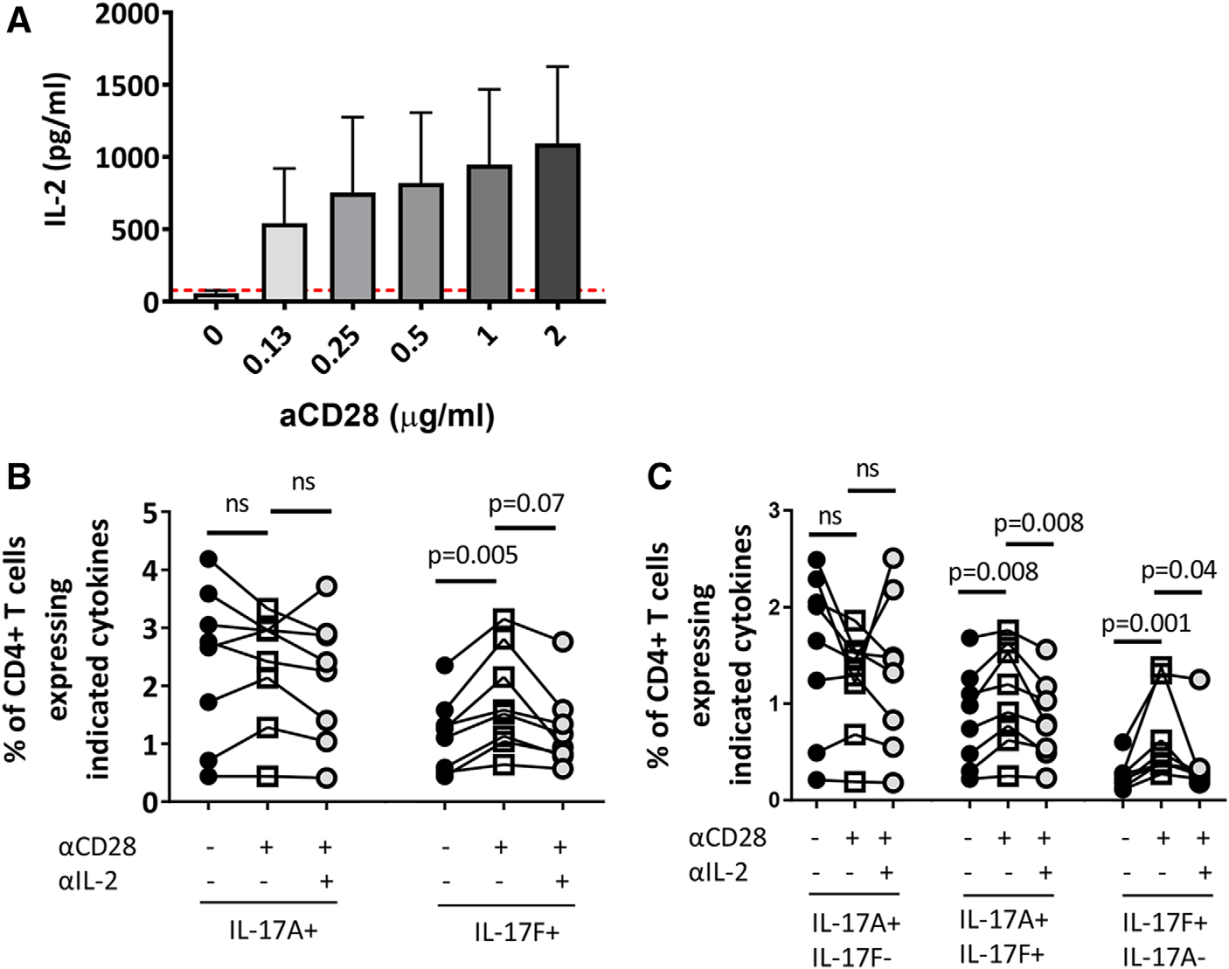
CD28‐driven induction of IL‐17F^+^ CD4^+^ T cells is mediated in part by IL‐2. Bulk CD4^+^ T cells were cultured with anti‐CD3 mAb, IL‐23, and IL‐1β in the presence of increasing doses of anti‐CD28 mAb (0–2 μg/mL). (A) After 3 days, culture supernatant was collected and levels of IL‐2 protein were analysed by ELISA (cumulative data from three independent experiments from *n* = 4 donors in total). Data are shown as the mean ± SEM. Dashed red line indicates the lower ELISA detection limit, respectively. (B) Bulk CD4^+^ T cells were cultured with anti‐CD3 mAb, IL‐23, and IL‐1β in the absence or presence of anti‐CD28 mAb and anti‐IL‐2 mAb for 3 days. Graphs show cumulative data of the frequencies of total IL‐17A^+^ and IL‐17F^+^ CD4^+^ T cells (B) or IL‐17A^+^IL‐17F^−^, IL‐17A^+^IL‐17F^+^, and IL‐17F^+^IL‐17A^−^ CD4^+^ T cells (C) (data from five independent experiments using *n* = 8 donors in total). Statistical analysis was performed using Friedman test with comparison to all samples by Dunn's Multiple Comparisons test.

Given that CD28 signaling is a strong enhancer of IL‐2 production by CD4^+^ T cells 26, we hypothesized that the CD28‐driven increase in IL‐17F^+^ CD4^+^ T cells was mediated by IL‐2. As expected, titration of anti‐CD28 mAb led to a dose‐dependent increase in IL‐2 in culture supernatants (Fig. 3A). To assess the role of IL‐2 in promoting IL‐17F^+^ CD4^+^ T cells, CD4^+^ T cell cultures derived from healthy peripheral blood were stimulated with anti‐CD3 mAb, anti‐CD28 mAb, IL‐1β, and IL‐23, in the absence or presence of neutralizing anti‐IL‐2 mAb. No significant changes in total IL‐17A^+^ CD4^+^ T cell frequencies were observed in the absence or presence of anti‐CD28 mAb with or without IL‐2 blocking antibody (Fig. 3B). In contrast, and as we observed in Fig. 2, addition of anti‐CD28 mAb promoted total IL‐17F^+^ CD4^+^ T cell frequencies (approximately 1% vs. 1.7% in absence vs. presence of anti‐CD28 mAb), and this effect was in part abrogated by IL‐2 blockade. Similarly, IL‐2 blockade significantly reduced the anti‐CD28 mAb mediated increase in IL‐17A^+^IL‐17F^+^ and IL‐17F^+^IL‐17A^−^ CD4^+^ T cells (Fig. 3C). Addition of an isotype control Ab did not affect the percentage of CD4^+^ T cells expressing IL‐17A or IL‐17F (Supporting Information Fig. 6). Collectively, these data indicate that CD28 signaling promotes IL‐17F expression in CD4^+^ T cells, which is in part mediated via an IL‐2 dependent mechanism.

### Investigating the effect of IL‐17A and IL‐17F addition versus blockade on synovial fibroblasts

**Figure 4:**
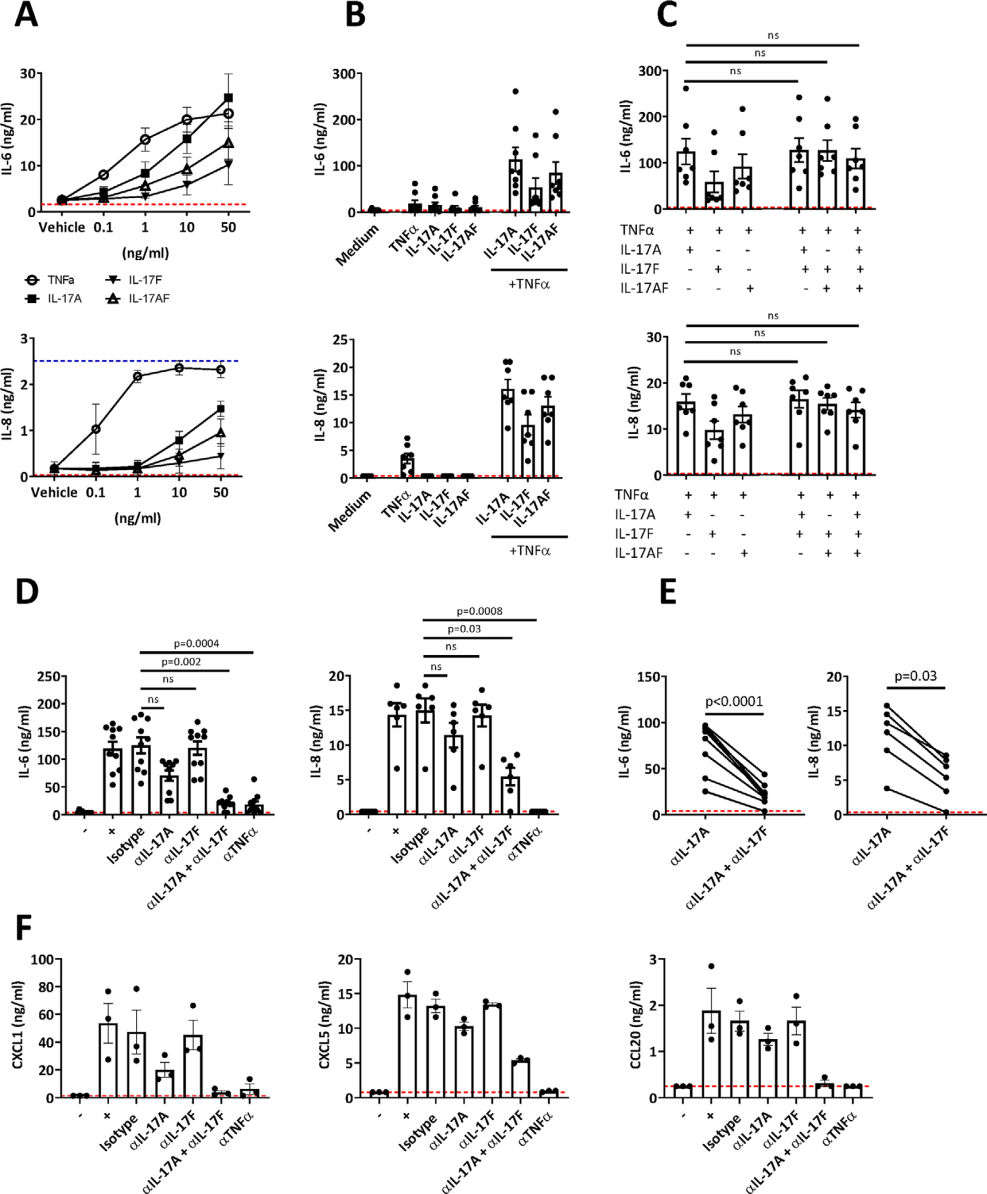
Investigating the effect of IL‐17A and IL‐17F addition versus blockade on synovial fibroblasts. (A) Synovial fibroblasts were cultured with indicated concentrations of IL‐17A, IL‐17F, and TNF‐α. The levels of IL‐6 and IL‐8 present in the fibroblast supernatant were measured by ELISA (cumulative data from four independent experiments using in total *n* = 3 RA fibroblasts and *n* = 1 PsA fibroblasts). Data are shown as the mean ± SEM. (B and C) Combinations of the above cytokines (IL‐17 cytokines at 10 ng/mL and TNF‐α at 1 ng/mL) were added to synovial fibroblasts. After 24 h incubation, supernatants were collected and IL‐6 (cumulative data from six independent experiments using in total *n* = 3 RA fibroblasts and *n* = 5 PsA fibroblasts) and IL‐8 (cumulative data from five independent experiments using in total *n* = 2 RA fibroblasts and *n* = 5 PsA fibroblasts) secretion levels were measured by ELISA. Bars show mean ± SEM of results. Statistical analysis was performed using Friedman test with comparison to IL‐17A and TNF‐α by Dunn's Multiple Comparisons test. (D–F) Synovial fibroblasts were cultured with a cocktail of human recombinant IL‐17A, IL‐17F, IL‐17AF, and TNF‐α (all at 10 ng/mL) in the absence or presence of the indicated neutralizing antibodies (1 μg/mL). After 24 h incubation, supernatants were collected and IL‐6 (cumulative data from 10 independent experiments using in total *n* = 4 RA fibroblasts and *n* = 6 PsA fibroblasts) and IL‐8 (cumulative data from four independent experiments using in total *n* = 3 RA fibroblasts and *n* = 3 PsA fibroblasts) secretion levels were measured by ELISA. In general, two different RA and PsA cell lines were used in each experiment. (D) Bars show mean ± SEM of results. Statistical analysis was performed using Friedman test with comparison to isotype by Dunn's Multiple Comparisons test. (E) Analysis of IL‐6 and IL‐8 protein levels conditioned either with αIL‐17A or αIL‐17A + αIL‐17F using a Wilcoxon‐matched pairs test. (F) Synovial fibroblast supernatants (one independent experiment using *n* = 2 PsA fibroblasts and *n* = 1 RA fibroblasts) were analyzed for levels of CXCL1, CXCL5, and CCL20 via Luminex. Bars show mean ± SEM of results. Dashed red and blue lines indicate lower and upper ELISA detection limits, respectively.

To assess the functional role of IL‐17F, RA and PsA fibroblasts were incubated for 24 h with human recombinant IL‐17A, IL‐17F, IL‐17AF, TNF‐α, or vehicle controls at the indicated concentrations. Recombinant IL‐17A, IL‐17F, IL‐17AF, or TNF‐α all induced a dose‐dependent increase in IL‐6 and IL‐8 secretion by fibroblasts (Fig. 4A). In agreement with literature, a hierarchy in inflammatory potency of the IL‐17 cytokines was observed: IL‐17A elicited higher levels of IL‐6 and IL‐8 in comparison to IL‐17F, while IL‐17AF induced intermediate IL‐6 and IL‐8 levels. In general, TNF‐α induced higher levels of IL‐6 and IL‐8, although at 50 ng/mL IL‐17A and TNF‐α induced similar levels of IL‐6. We investigated whether combinations of IL‐17A, IL‐17F, IL‐17AF, and TNF‐α further enhanced the secretion of inflammatory cytokines. The combination of IL‐17A and TNF‐α resulted in a synergistic increase in IL‐6 and IL‐8 secretion compared with addition of IL‐17A or TNF‐α alone (Fig. 4B). Similarly, synergistic responses were observed between TNF‐α and IL‐17F, and TNF‐α and IL‐17AF. Addition of IL‐17F or IL‐17AF to IL‐17A and TNF‐α did not lead to further additive or synergistic effects on IL‐6 or IL‐8 secretion by fibroblasts (Fig. 4C).

To further investigate the functional impact of an environment in which both IL‐17A and IL‐17F are present, we assessed the effect of dual IL‐17A and IL‐17F blockade in this reductionist system. IL‐17A, IL‐17F, IL‐17AF, and TNF‐α were added to RA or PsA synovial fibroblasts in the presence of IL‐17A, IL‐17F, or dual IL‐17A/IL‐17F blocking antibodies. Anti‐TNF‐α mAb and human IgG1 isotype control were added as positive and negative control, respectively. As before, addition of the cytokine cocktail led to an increase in IL‐6 and IL‐8 production by fibroblasts in comparison to medium alone. Addition of isotype control or anti‐IL‐17F blocking antibody had no effect on IL‐6 or IL‐8 production. In contrast, IL‐6 and IL‐8 secretion was reduced upon addition of IL‐17A blocking antibody, and cytokine production was further reduced upon combined blockade of IL‐17A and IL‐17F using bimekizumab (Fig. 4D). When compared directly, dual IL‐17A and IL‐17F blockade reduced IL‐6 and IL‐8 levels significantly more than IL‐17A blockade alone (Fig. 4E). TNF‐α blockade reduced IL‐6 to similar levels as bimekizumab but was more effective at reducing IL‐8 levels. We also measured the levels of additional pro‐inflammatory mediators, CXCL1, CXCL5, and CCL20 in the fibroblast supernatant by Luminex (Fig. 4F). Similar to IL‐6 and IL‐8, dual neutralization of IL‐17A and IL‐17F reduced the production of these chemokines by fibroblasts further than IL‐17A blockade alone. Collectively, these data suggest that a combined blockade of IL‐17A and IL‐17F is more effective at reducing inflammation than blockade of IL‐17A alone.

### IL‐17A and IL‐17F expressing CD4^+^ T cells display different cytokine profiles

**Figure 5:**
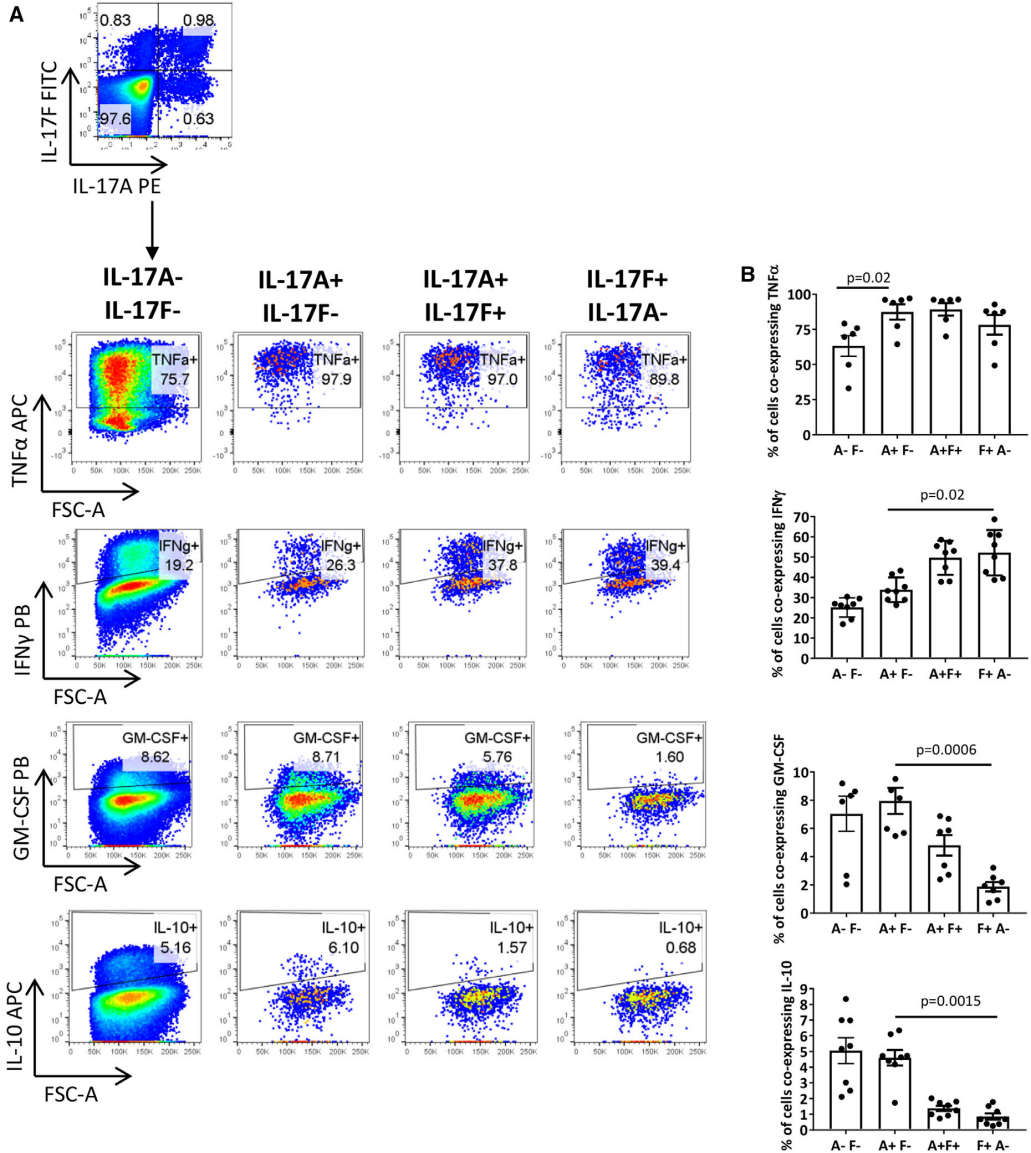
IL‐17F^+^IL‐17A^−^ and IL‐17A^+^IL‐17F^+^ CD4^+^ T cells display a different cytokine profile in comparison to IL‐17A^+^IL‐17F^−^ CD4^+^ T cells. Bulk CD4^+^ T cells were cultured with anti‐CD3 mAb, anti‐CD28 mAb, IL‐23, and IL‐1β. After 3 days, cells were re‐stimulated and assessed for intracellular cytokine expression by flow cytometry. (A) Representative dot plot of IL‐17A^+^ and IL‐17F^+^ CD4^+^ T cell populations. Following gating within IL‐17A^−^IL‐17F^−^, IL‐17A^+^IL‐17F, IL‐17A^+^IL‐17F^+^, and IL‐17F^+^IL‐17A^−^ CD4^+^ T cells, the co‐expression of other cytokines was analyzed. (B) Cumulative data showing the percentage of each population co‐expressing TNF‐α (four independent experiments using *n* = 6 donors in total), IFN‐γ (five independent experiments using *n* = 8 donors in total), GM‐CSF (three independent experiments using *n* = 7 donors in total), or IL‐10 (six independent experiments using *n* = 8 donors in total). Bars represent the mean ± SEM. Statistical analysis was performed using Friedman test with comparison to IL‐17A^+^IL‐17F^−^ CD4^+^ T cells by Dunn's Multiple Comparisons test.

We next assessed the co‐expression of TNF‐α, IFN‐γ, GM‐CSF, and IL‐10 in IL‐17A^+^ and IL‐17F^+^ populations upon 3‐day stimulation of bulk CD4^+^ T cells with anti‐CD3 mAb, anti‐CD28 mAb, IL‐1β, and IL‐23. IL‐17A^+^IL‐17F^−^, IL‐17A^+^IL‐17F^+^, and IL‐17F^+^IL‐17A^−^ CD4^+^ T cells displayed similarly high frequencies of cells expressing TNF‐α (Fig. 5A and B). When compared to IL‐17A^+^IL‐17F^−^ CD4^+^ T cells, IL‐17F^+^IL‐17A^−^ CD4^+^ T cells displayed significantly higher frequencies of IFN‐γ expressing cells (approximately 34% vs. 52%, respectively) and significantly lower frequencies of IL‐10 expressing cells (approximately 4.6% vs. 0.9%, respectively). IL‐17A^+^IL‐17F^−^ displayed a significantly higher percentage of cells co‐expressing GM‐CSF versus IL‐17F^+^IL‐17A^−^ (approximately 8% vs. 1.9%, respectively). IL‐17A^+^IL‐17F^+^ CD4^+^ T cells displayed intermediate co‐expression levels for IFN‐γ, IL‐10, and GM‐CSF (approximately 50%, 1.4% and 4.8%, respectively). These data indicate that IL‐17A^+^ and IL‐17F^+^ CD4^+^ T cells display different cytokine profiles.

We further investigated whether IL‐17F^+^ CD4^+^ T cells show a Th1 bias by comparing the expression of the transcription factor T‐bet, in IL‐17F^+^ versus IL‐17A^+^ CD4^+^ T cells. However, our data showed no consistent difference in the expression of T‐bet across the different IL‐17 populations (Supporting Information Fig. 7).

### Presence of IL‐17F^+^ CD4^+^ T cells in inflammatory arthritis

**Figure 6:**
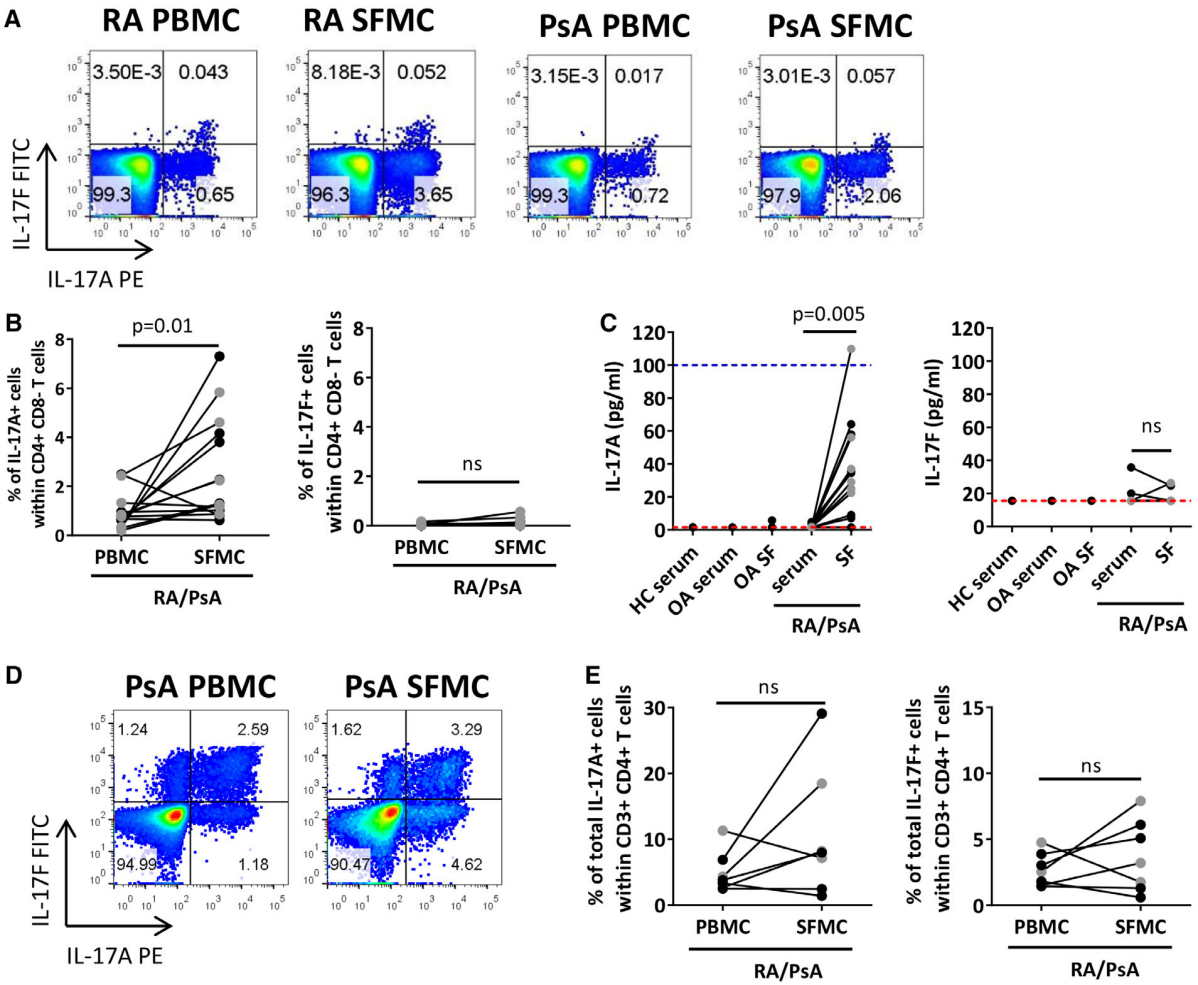
Investigating the presence of IL‐17A^+^ and IL‐17F^+^ CD4^+^ T cells in inflammatory arthritis. (A–C) Matched PBMC and SFMC from RA and PsA donors were stimulated for 3 h with PMA and ionomycin and then assessed for intracellular IL‐17A and IL‐17F cytokine expression by flow cytometry. Representative dot plots (A) and cumulative data (B) show frequencies of IL‐17A and IL‐17F expressing cells within the total CD4^+^ T cell population of matched PBMC and SFMC from RA (black symbols) and PsA (grey symbols). Data show 12 independent experiments using *n* = 8 RA donors and *n* = 6 PsA donors in total. (C) In one independent experiment, serum from healthy controls (*n* = 2) and paired serum and synovial fluid from OA patients (*n* = 2), RA patients (*n* = 7), and PsA patients (*n* = 6) were analyzed by ELISA for protein levels of IL‐17A and IL‐17F. Dashed red and blue lines represent the lower and upper ELISA detection limits, respectively. (D and E) Paired PBMC and SFMC samples from patients with RA (black symbols) or PsA (grey symbols) were cultured with plate‐bound anti‐CD3 mAb, anti‐CD28 mAb, IL‐1β, and IL‐23. After 3 days incubation, cells were re‐stimulated for 3 h with PMA and ionomycin. Representative dot plots and **(D)** cumulative data **(E)** showing the presence of IL‐17A or IL‐17F expressing cells. Data show 6 independent experiments with *n* = 4 RA donors and *n* = 3 PsA donors in total. Data were analyzed using Wilcoxon matched‐pairs test.

Our final aim was to investigate the presence of IL‐17F^+^ CD4^+^ T cells and IL‐17F protein in the blood and inflamed joints of patients with RA or PsA. Paired peripheral blood mononuclear cells (PBMCs) and synovial fluid mononuclear cells (SFMCs) from RA or PsA patients were stimulated ex vivo with PMA/ionomycin. When gated within CD3^+^ CD4^+^ T cells, IL‐17A expressing cells were increased in the synovial fluid (SF) compared to the peripheral blood (PB) of patients with inflammatory arthritis (IA). In contrast, very few IL‐17F^+^ CD4^+^ T cells were detected in matched PB and SF samples from IA patients (Fig. 6A and B). We investigated the presence of IL‐17A and IL‐17F protein in paired serum and SF from patients with IA, and in serum from healthy controls and serum and SF from osteoarthritis patients as controls. In line with previous studies 23, 27, IL‐17A protein was detected at increased concentrations in the SF versus serum of IA patients, while no IL‐17A protein was detectable in healthy control serum or paired serum or SF from patients with osteoarthritis. In contrast, IL‐17F was undetectable or present at very low levels in both serum and SF samples (Fig. 6C). Increasing the ex vivo stimulation with either PMA and ionomycin or anti‐CD3 mAb and anti‐CD28 mAb did not reveal any significant increase in IL‐17F^+^ CD4^+^ T cell frequencies (*n* = 2, data not shown).

Although we did not detect IL‐17F, we did detect IL‐1β and IL‐23 at elevated levels in the majority of SF versus serum samples of patients with RA or PsA (Supporting Information Fig. 8). We tested whether stimulating RA or PsA PBMC and SFMC in vitro with anti‐CD3 and CD28 mAbs in the presence of IL‐1β and IL‐23 would induce IL‐17F^+^ CD4^+^ T cells. Culturing PBMC and SFMC under these conditions revealed clearly detectable frequencies of IL‐17F expressing CD4^+^ T cells (both single and double positive; Fig. 6D and E). There was no consistent difference in the frequencies of IL‐17A or IL‐17F‐expressing CD4^+^ T cells between cells derived from the synovial fluid or the peripheral blood.

## Discussion

Our findings demonstrate that high‐strength T cell stimulation drives IL‐17F expressing CD4^+^ T cells and that IL‐17A and IL‐17F are differentially regulated. In line with previous studies, we show that the pro‐inflammatory functions of IL‐17F are enhanced in the presence of TNF‐α and that in an environment in which IL‐17A, IL‐17F, and TNF‐α are present, dual IL‐17A and IL‐17F blockade is more effective at reducing inflammatory cytokine production than blockade of IL‐17A alone.

Expanding upon our previous work 3, 4, 5, we show that LPS addition to CD4^+^ T cell/ CD14^+^ monocyte co‐cultures leads to an increase in the percentage of both IL‐17A^+^ and IL‐17F^+^ CD4^+^ T cells. Although in this study the mechanism by which LPS‐activated monocytes induce IL‐17F^+^ CD4^+^ T is not fully elucidated, we show that IL‐1β and IL‐23 are likely to play a role. When comparing induction of IL‐17F^+^ CD4^+^ T cells by LPS‐activated monocytes versus soluble anti‐CD28 mAb and recombinant IL‐1β and IL‐23, we observed that while both cultures induced IL‐17A^+^IL‐17F^+^ CD4^+^ T cells, only the latter culture resulted in IL‐17F^+^IL‐17A^−^ CD4^+^ T cells. We postulate that the anti‐CD28 mAb elicits a stronger co‐stimulation signal than the LPS‐activated monocytes, which drives the induction of IL‐17F^+^IL‐17A^−^ CD4^+^ T cells. Indeed, we demonstrate that addition of anti‐CD28 mAb to CD4^+^ T cell cultures significantly increases the frequency of IL‐17F^+^ CD4^+^ T cells. Furthermore, we show that high‐strength rather than low strength anti‐CD3 stimulation favors induction of IL‐17F^+^ CD4^+^ T cells. These data indicate that high‐strength T cell stimulation drives the induction of human IL‐17F^+^ CD4^+^ T cells. To the best of our knowledge, this has not been previously reported. It has been shown that high‐strength stimulation decreased IL‐17A^+^ CD4^+^ T cell frequency 7. Additionally, a mouse study demonstrated that anti‐CD28 mAb suppressed the differentiation of IL‐17A^+^ CD4^+^ T cells in an IL‐2 and IFN‐γ dependent mechanism 6. Under our culture conditions, we did not observe a consistent decrease in the frequency of total IL‐17A^+^ CD4^+^ T cells upon addition of higher doses of anti‐CD28 mAb or anti‐CD3 mAb.

We demonstrate that the anti‐CD28 mAb mediated induction of human IL‐17F^+^ CD4^+^ T cells is in part mediated by IL‐2. In contrast, IL‐2 blockade did not lead to consistent differences in the frequency of human IL‐17A^+^ CD4^+^ T cells. Differential effects of IL‐2 on IL‐17A and IL‐17F regulation have been previously reported in mice 28, however the results differ to those reported here. Using mouse cells, Yang et al. demonstrated that in the presence of high levels of IL‐6, IL‐2 addition decreased the percentage of IL‐17A^+^ CD4^+^ T cells, while not affecting the frequency of IL‐17F^+^ CD4^+^ T cells 28. While no further studies report on the effect of IL‐2 on IL‐17F^+^ CD4^+^ T cell induction, numerous mouse studies demonstrate that IL‐2 downregulates IL‐17A^+^ CD4^+^ T cells 6, 28, 29, 30, 31. One explanation for our contrasting results could be that IL‐2 affects IL‐17A and IL‐17F in humans differently. Furthermore, we assessed the effect of IL‐2 on IL‐17A^+^ CD4^+^ T cells induced from bulk CD4^+^ T cell cultures, while the mouse studies used naïve CD4^+^ T cell polarization protocols 28, 29. Our data indicate that IL‐17A and IL‐17F are differentially regulated, at least in vitro, thus supporting previous evidence of differential regulation of IL‐17A and IL‐17F 32, 33.

Our findings show that IL‐17A^+^IL‐17F^−^, IL‐17A^+^IL‐17F^+^, and IL‐17F^+^IL‐17A^−^ CD4^+^ T cells display different cytokine profiles; IL‐17F^+^IL‐17A^−^ (and to a lesser extent IL‐17F^+^IL‐17A^+^) CD4^+^ T cells displayed lower frequencies of IL‐10 and GM‐CSF and higher frequencies of IFN‐γ when compared with IL‐17A^+^IL‐17F^−^ CD4^+^ T cells. To the best of our knowledge, the cytokine profile of the different IL‐17A/IL‐17F CD4^+^ T cell populations has not been characterized previously. Aschenbrenner et al. recently performed RNA‐seq on human Th17‐IL‐10^−^ and Th17‐IL‐10^+^ clones. Interestingly, *IL17F* and *IFNG* were highly expressed in Th17‐IL‐10^−^ cells associated with a "pathogenic" Th17 subtype, whereas *IL17A* was preferentially expressed in Th17‐IL‐10^+^ cells associated with a "nonpathogenic" Th17 phenotype. Functionally, Th17‐IL‐10^−^ cells had the ability to polarise monocytes into an inflammatory M1 phenotype, while Th17^−^IL‐10^+^ cells induced an M2 anti‐inflammatory phenotype 34. Collectively, our work and other studies 11, 34 highlight that performing molecular and functional characterization of isolated IL‐17A/IL‐17F CD4^+^ T cell sub‐populations would be an interesting avenue to pursue.

Our data show, in line with other studies 14, that IL‐17F induces IL‐6 and IL‐8 secretion from synovial fibroblasts that is enhanced in the presence of TNF‐α. However, in comparison with IL‐17A and IL‐17AF, IL‐17F is the least potent. Our blocking experiments revealed that while blockade of IL‐17F alone has no effect on cytokine secretion, dual IL‐17A and IL‐17F blockade by bimekizumab is more effective at reducing IL‐6, IL‐8, CXCL1, CXCL5, and CCL20 secretion by fibroblasts compared to blockade of IL‐17A alone. These data show that in the presence of IL‐17A, IL‐17F has a limited effect on fibroblasts. However, in the absence of IL‐17A, IL‐17F can synergize with TNF‐α and elicit a significant inflammatory response. Collectively, our data support previous studies 19, 20 and demonstrate that in an environment in which both IL‐17A and IL‐17F are present, dual IL‐17A and IL‐17F blockade may reduce inflammation to a greater extent. Recent clinical studies have shown that dual IL‐17A and IL‐17F blockade by bimekizumab results in rapid and profound efficacy in the treatment of PsA 20 and psoriasis 21. However, in the absence of data from direct head‐to‐head studies, it remains to be concluded whether in these diseases there is a clinically superior effect of dual IL‐17A and IL17F blockade versus blockade of IL‐17A alone.

In agreement with previous studies 22, 23, 24, 27, we demonstrate the presence of IL‐17A^+^ CD4^+^ T cells and IL‐17A protein at elevated levels in the synovial fluid versus blood from patients with RA or PsA. In contrast, few IL‐17F^+^ CD4^+^ T cells were detected in the PB or SF of inflammatory arthritis patients, and ELISA results showed IL‐17F to be undetectable or present at very low levels in inflammatory arthritis paired serum and SF samples. Current literature shows discrepancies regarding the presence of IL‐17F in SF from inflammatory arthritis patients 18, 35. In addition, a recent report by Kolbinger et al. detected levels of IL‐17F and IL‐17A in the serum of PsA, AS, and psoriasis patients using a highly sensitive Singulex immunoassay. That study suggested IL‐17F is present at higher levels than IL‐17A 36. Inconsistencies across studies in levels of IL‐17F levels detected in patient samples may be due to differences in the specific assay method, differences in sampling preparation or heterophilic antibody interference 37. In summary, while our data easily detect IL‐17A^+^ CD4^+^ T cells and IL‐17A protein in inflammatory arthritis SF, we do not confirm the presence of IL‐17F^+^ CD4^+^ T cells or IL‐17F protein in inflammatory arthritis. However, our in vitro stimulation cultures demonstrate that CD4^+^ T cells from the PB and SF of patients with inflammatory arthritis have the capacity to express IL‐17F given a suitable environment.

Collectively, our data indicate that high‐strength T cell stimulation induces IL‐17F expressing CD4^+^ T cells and that IL‐17A and IL‐17F are differentially regulated. Moreover, we show that the cytokine profile of IL‐17F^+^ CD4^+^ T cells differs from IL‐17A^+^ CD4^+^ T cells, highlighting the need to further characterize these IL‐17‐expressing sub‐populations. Finally, our data demonstrate that dual neutralization of IL‐17A and IL‐17F may be more effective at reducing inflammation than blockade of IL‐17A alone. This may be beneficial in alleviating symptoms of inflammatory diseases in which both IL‐17A and IL‐17F are present.

## Materials and methods

### Cell isolation

Peripheral blood samples were collected from healthy adult volunteers and peripheral blood or synovial fluid samples from patients with RA or PsA attending the Guy's Hospital Rheumatology Department (Research Ethics Committee Ref 06/Q0705/20). Patient cohort information is provided in Supporting Information Tables 1 and 2. PBMCs or SFMCs were isolated via density gradient centrifugation using Lymphoprep (Axis‐Shield, Oslo, Norway). CD14^+^ monocytes were isolated by positive selection using CD14 microbeads (Miltenyi Biotec, Bergisch‐Gladbach, Germany; average purity 95%) and CD4^+^ T cells were isolated by negative depletion (Miltenyi Biotec; average purity 97%).

RA and PsA fibroblast cell lines were either generated in‐house or received as a kind gift from Dr. Anca Catrina (Karolinska University Hospital, Sweden). In‐house fibroblast cell lines were obtained from RA or PsA synovial membrane tissue samples, collected via arthroscopic biopsy from the joint (REC refs 17/LO/1940 and 07/H0809/35). Synovial tissue samples were cut into 1mm^3^ pieces and individually incubated at 37**°**C with 5% CO_2_ in a well coated with 0.1% bovine gelatin (Sigma–Aldrich, Missouri, USA) and supplemented with DMEM (ThermoFisher Scientific, Massachusetts, USA) with 20% FCS, 100 units/mL penicillin, 100 μg/mL of streptomycin, 2% glutamine, and fungizone (all ThermoFisher Scientific). Tissue explants were cultured until synovial fibroblasts had grown out of the synovial tissue. Cells were passaged when they reached 80% confluence in T175 flasks. Fibroblast lines were used between passage 2 and 8.

### Cell‐free serum and synovial fluid samples

For serum samples, blood was collected in a no additive serum tube (Fisher Scientific, Loughborough, UK) and stored at 4**°**C for 1 h before centrifugation at 1200 rpm for 10 min and serum harvesting. Synovial fluid was centrifuged for 10 min at 1200 rpm after which the cell‐free fluid was removed. Serum from healthy controls (*n* = 2) and paired serum and synovial fluid from OA patients (*n* = 2), RA patients (*n* = 7), and PsA patients (*n* = 6) were stored at −80**°**C.

### CD4^+^ T cell or mononuclear cell cultures

Human PBMCs or SFMCs were cultured in Roswell Park Memorial Institute media (RPMI) 1640 (Gibco, Invitrogen, UK) supplemented with 10% FCS, 100 units/mL penicillin, 100 μg/mL of streptomycin, and 1% glutamine. Freshly isolated bulk CD4^+^ T cells (0.5 × 10^6^) were co‐cultured for 3 days at a 1:1 ratio with autologous CD14^+^ monocytes in a 48‐well plate with 100 ng/mL soluble anti‐CD3 mAb (clone OKT3, Janssen Cilag, High Wycombe, UK or Biolegend, Cambridge, UK) in the absence or presence of 100 ng/mL LPS (Sigma–Aldrich). In some experiments, 1 × 10^6^ isolated CD4^+^ T cells were cultured with plate‐bound anti‐CD3 mAb (1.25 μg/mL) in the absence or presence of soluble anti‐CD28 mAb (1 μg/mL, R&D Systems, Minneapolis, USA), human recombinant IL‐1β (10 ng/mL, Peprotech, London, UK) and IL‐23 (20 ng/mL, R&D, UK) with or without neutralizing anti‐human IL‐2 mAb (1 μg/mL, goat IgG, R&D) or isotype control mAb (goat IgG, BD Biosciences, San Jose, USA). Some experiments involved titrating soluble anti‐CD28 mAb (0–2 μg/mL) or plate‐bound anti‐CD3 mAb (0–5 μg/mL). For PBMC and SFMC cultures, 1 × 10^6^ cells were cultured with plate‐bound anti‐CD3 and soluble anti‐CD28 mAb in the absence or presence of IL‐1β (10 ng/mL) and IL‐23 (20 ng/mL).

### Fibroblast cultures

For fibroblast assays, 10 × 10^3^ fibroblasts were seeded per well in a 96 well flat‐bottom plate in DMEM culture medium and incubated for 24 h at 37 **°**C with 5% CO_2_. Following supernatant removal, recombinant human cytokines IL‐17A, TNF‐α (both from Miltenyi Biotec), IL‐17F, or IL‐17AF (both from R&D) were added either individually or in combination at the indicated concentrations. After 24 h, fibroblast supernatants were collected and protein levels analyzed by ELISA. Blocking antibodies (1 μg/mL) included anti‐IL‐17A mAb (targeting both the IL‐17A homodimer and IL‐17AF heterodimer), anti‐IL‐17F (targeting the IL‐17F homodimer only), and bimekizumab (targeting IL‐17A, IL‐17F, and IL‐17AF; all human IgG). These IL‐17 blocking antibodies were generated in‐house by UCB Pharma and characterized previously 20. For TNF‐α blockade, adalimumab (human IgG1, Abbott Laboratories, Chicago, IL, USA) was used. Human IgG1 (UCB in‐house) was used as isotype control.

### Flow cytometry

For ex vivo analysis, 1 × 10^6^ PBMC, SFMC, or CD4^+^ T cells were cultured for 3 h in the presence of PMA; 50 ng/mL, Sigma–Aldrich), ionomycin (750 ng/mL, Sigma–Aldrich), and Golgi‐Stop (monensin, according to manufacturer's guidelines, BD Biosciences). Similarly, following in vitro culture, cells were stimulated for 3 hours with PMA, ionomycin and Golgi‐Stop. In some in vitro experiments, PMA (10 ng/mL), ionomycin (1 μg/mL), and brefeldin (10 μg/mL) (Sigma–Aldrich) were used. To gate for live CD4^+^ T cells, cells were labeled with a fixable viability dye (eFluor506 or eFluor780, eBioscience, San Diego, CA, USA), anti‐CD14 and/or CD8 mAbs before fixing in 2% paraformaldehyde (Merck & Co.,Inc., Kenilworth, NJ, USA), and permeabilization with 0.5% saponin (Sigma–Aldrich). Cells were then stained intracellularly with combinations of antibodies (Supporting Information Table 3). Cells were acquired using a FACSCantoII or LSR Fortessa (BD Biosciences). Flow cytometry data were analyzed using FlowJo software (Tree Star, Inc., Ashland, OR, USA). Cytokine co‐expression analysis was performed only when the event count within the IL‐17A^+^ or IL‐17F^+^ population exceeded 300. All experiments adhered to guidelines for the use of flow cytometry in immunological studies 38.

### Cytokine detection

Primary cell culture supernatants were analyzed using ELISA kits for IL‐17A (Biolegend or eBioscience), IL‐17F (eBioscience), IL‐2, or IFN‐γ (both from Biolegend). In some cases, cytokine levels were determined by Luminex assay (Bio‐Plex Pro^TM^ Human Th17 Cytokine Assays, Bio‐Rad Laboratories, CA, USA). Fibroblast culture supernatants were analyzed using standard IL‐6 and IL‐8 Biolegend ELISA kits and a luminex assay (R&D) for CXCL1, CXCL5, and CCL20 levels. Cell‐free serum and synovial fluid samples were analyzed using Platinum kits for IL‐17A and IL‐17F (eBioscience) and a Luminex assay (Bio‐Plex Pro^TM^ Human Th17 Cytokine Assays, Bio‐Rad Laboratories) for IL‐1β and IL‐23. All analyses were performed according to manufacturer's instructions.

### Statistical analysis

Statistical differences were calculated using Prism 7.0 software (Graphpad, San Diego, CA). A D'Agonisto and Pearson omnibus normality test was followed by statistical significance testing using the appropriate tests as described in the figure legends. Data sets with *n* values < 8 were tested non‐parametrically. *P*‐values < 0.05 were considered statistically significant.

## Conflict of interest

This study was supported in part by research support from UCB Pharma. A.M. and D.M. are employees of UCB Pharma and hold shares in UCB Pharma. L.A.B. is a recent employee of UCB Pharma. L.S.T. has received speaker fees and/or research support from GSK, Novo Nordisk A/S, UCB, and Novartis. B.W.K. has received research support from Eli‐Lilly, Novartis, Roche Pharmaceuticals, and UCB Pharma, and has been an advisor or received speaker fees from Eli‐Lilly, Janssen, and Novartis. A.C. declared no conflict of interest.

**Abbreviations**
AxSpA: axial spondylitis
CIA: collagen‐induced arthritis
FM: fluorescence minus control
OA: osteoarthritis
PsA: psoriatic arthritis
RA: rheumatoid arthritis

## Supporting information

Supporting Information
