# Supplementary material for: Presence, function, and regulation of IL‐17F‐expressing human CD4+ T cells
Source: Eur J Immunol. 2020 Jan 16;50(4):568–80. doi: 10.1002/eji.201948138 (PMC7187427; doi:10.1002/eji.201948138)
Supplement: Supplementary file 1 — Supporting Information [file EJI-50-568-s001.pdf]

European Journal of Immunology

Supporting Information
for
DOI 10.1002/eji.201948138

Lachrissa A. Burns, Ash Maroof, Diane Marshall, Kathryn J. A. Steel, Sylvine Lalnunhlimi, Suzanne Cole, Anca Catrina, Bruce Kirkham and Leonie S. Taams

Presence, function, and regulation of IL-17F-expressing human CD4^{+} T cells

© 2019 The Authors. *European Journal of Immunology* published by WILEY-VCH Verlag GmbH & Co. KGaA, Weinheim.

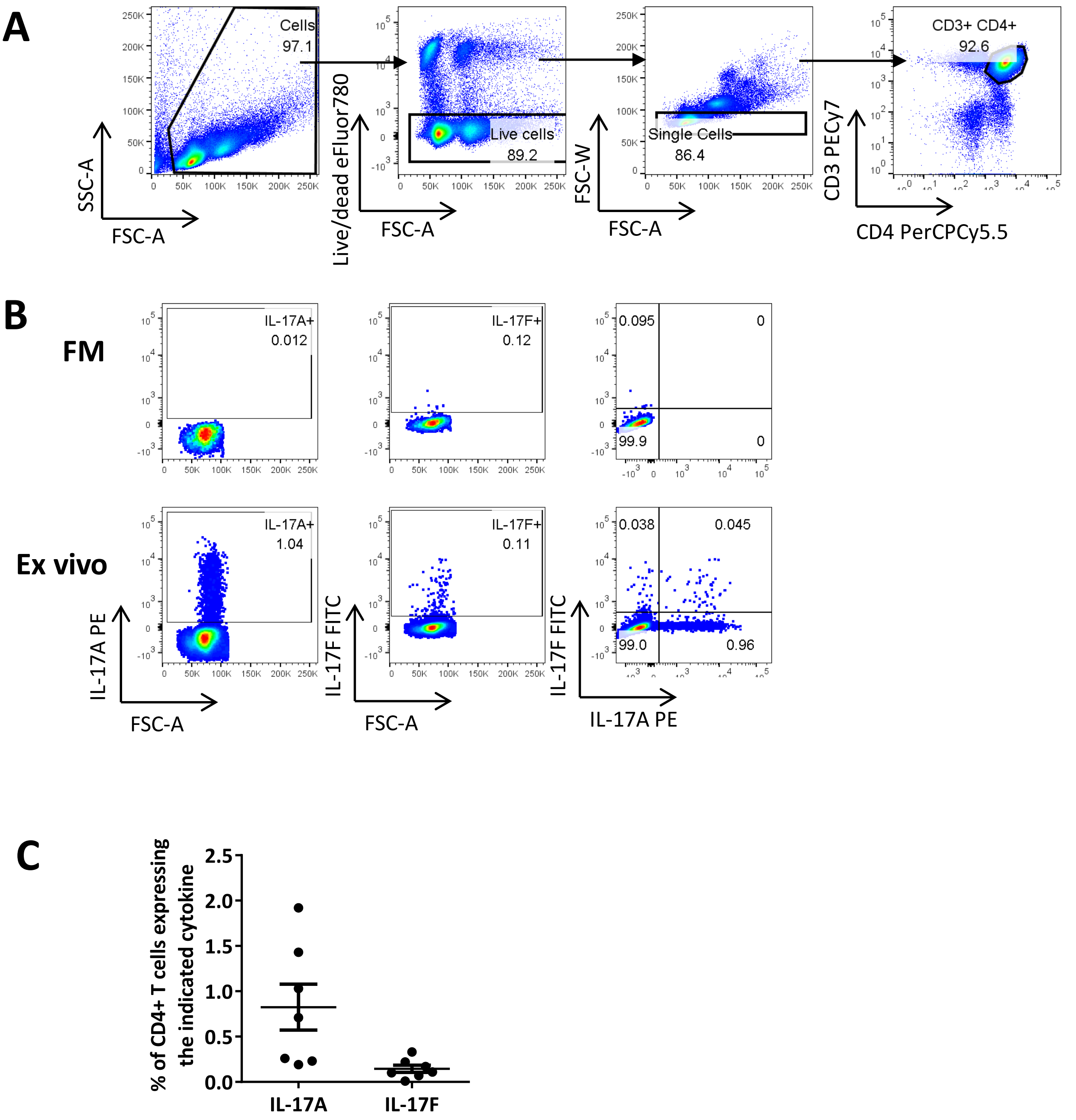

Supplementary Figure 1. Investigating the presence of IL-17A+ and IL-17F+ CD4+ T cells in *ex vivo* stimulated healthy control CD4+ T cells. Healthy donor bulk CD4+ T cells were stimulated for 3 hours with PMA, ionomycin and Golgistop and then assessed for intracellular IL-17A and IL-17F cytokine expression. **(A)** Flow cytometric gating strategy, **(B)** representative dot plots showing fluorescence minus (FM) controls and the frequencies of IL-17A and IL-17F expressing cells within the total CD4+ T cell population and **(C)** cumulative data for the frequency of IL-17A+ and IL-17F+ CD4+ T cells (n=7).

A

B

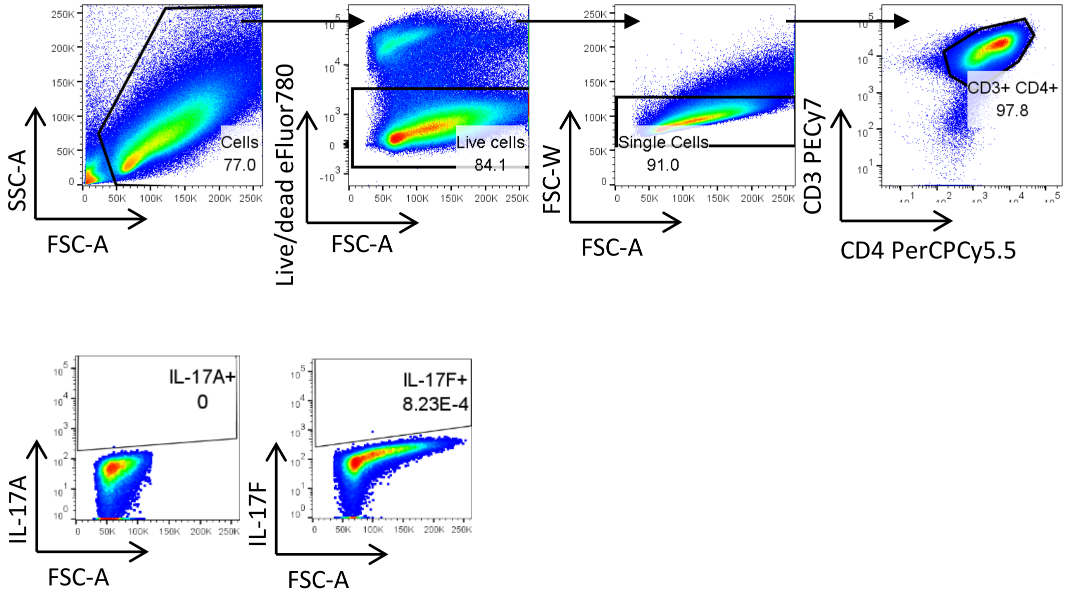

Supplementary Figure 2. Representative gating strategy and fluorescence minus controls for *in vitro* cultures. (A) Cells were gated for lymphocytes (SSC-A vs FSC-A). A 'dump' gate was then applied, whereby the viability dye and CD14 antibody were on the same fluorochrome, allowing for the simultaneous exclusion of dead and CD14+ cells. Next, a FSC-W/FSC-A gate was applied allowing the exclusion of doublet CD4+ T cells. CD3+ CD4+ T cells were then selected for analysis of cytokine expression. **(B)** Representative dot plots for cytokine fluorescence minus controls.

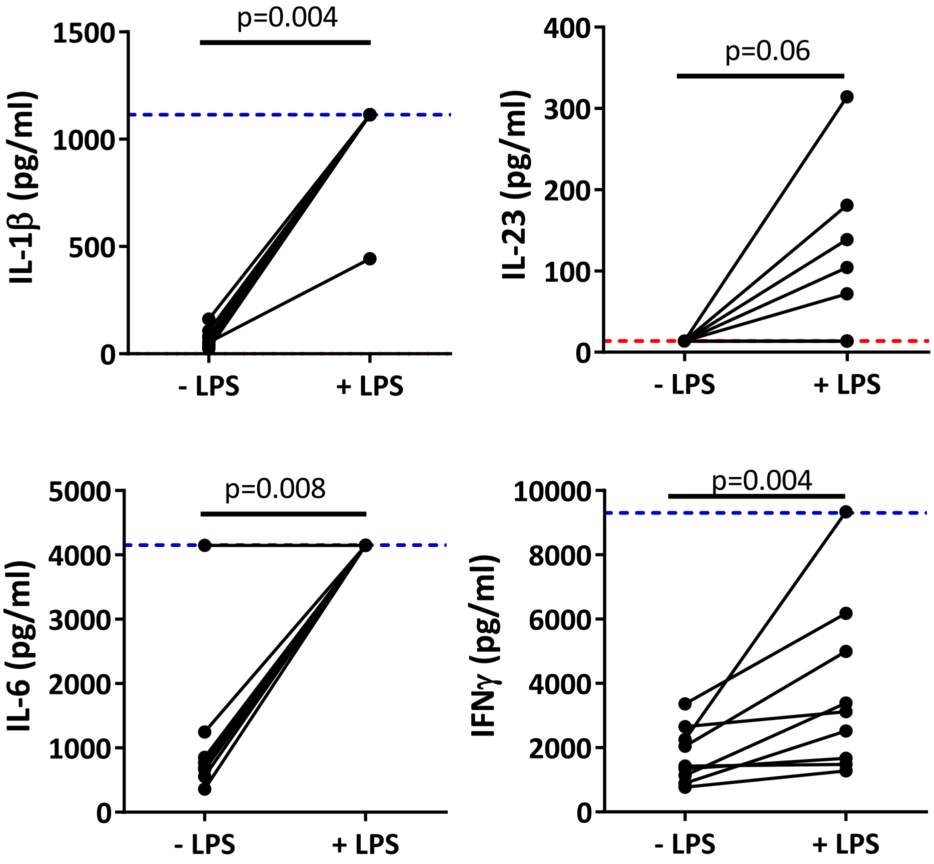

Supplementary Figure 3. Elevated levels of IL-1β, IL-6 and IFNγ are detected in CD4+ T cell / LPS-activated CD14+ monocyte co-cultures. Bulk CD4+ T cells from healthy donors (n=9) were co-cultured 1:1 with autologous monocytes and stimulated with anti-CD3 mAb (100 ng/ml) in the absence (control) or presence of LPS (100 ng/ml). Supernatants were collected and IL-1β, IL-23, IL-6 and IFNγ levels were detected by Luminex. Upper blue and lower red dashed lines indicate the upper and lower detection limits, respectively. Data were analysed using Wilcoxon matched-pairs signed rank test.

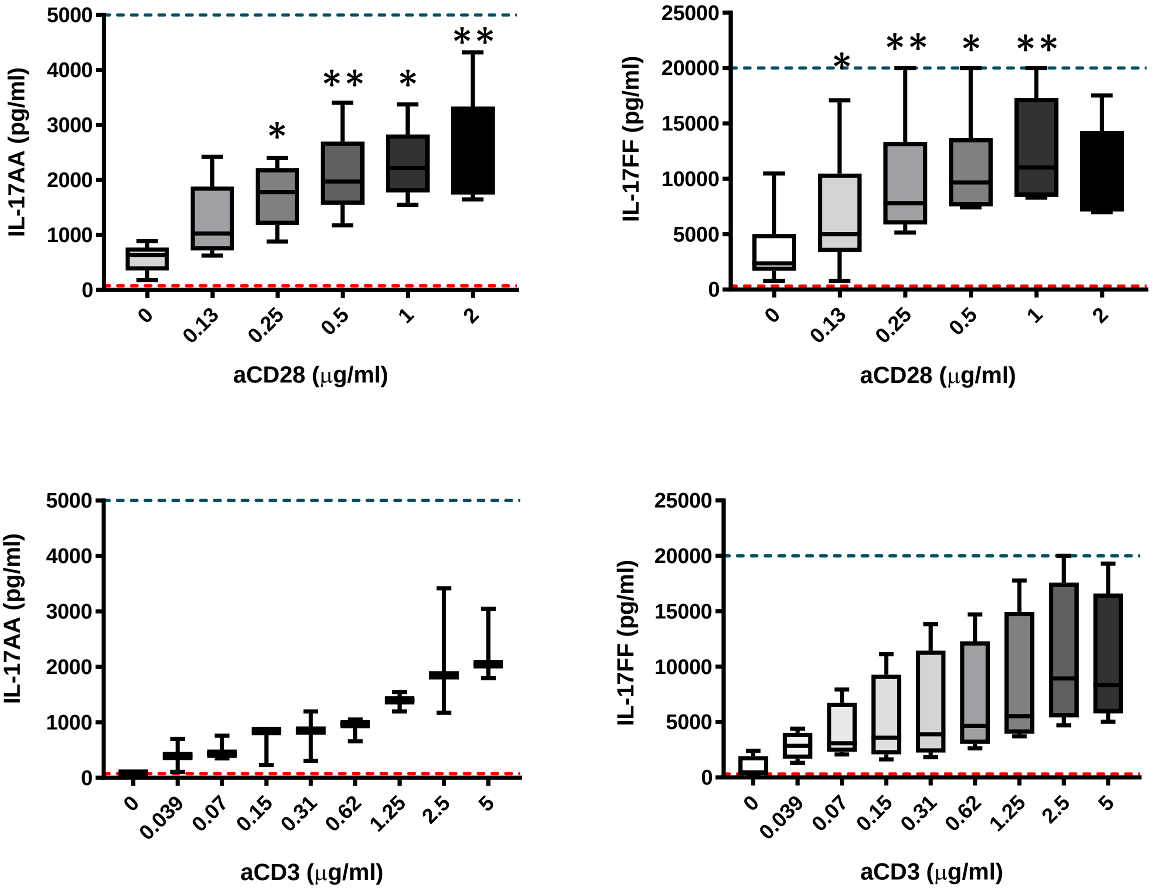

Supplementary Figure 4. IL-17A and IL-17F protein levels with increasing doses of anti-CD28 mAb or anti-CD3 mAb. Bulk CD4+ T cells ($0.2x10^6$) were cultured with plate-bound anti-CD3 mAb, IL-1β and IL-23 in the presence of increasing doses of soluble anti-CD28 mAb (0-2μg/ml) (n=5-6). **(B)** Bulk CD4+ T cells ($0.2x10^6$) were cultured with soluble anti-CD28 mAb, IL-1β and IL-23 in the presence of increasing doses of plate-bound anti-CD3 mAb (0-5μg/ml) (n=3-4). After 3 days, culture supernatants were collected and analysed for IL-17A and IL-17F protein expression via ELISA. Upper blue and red dashed lines indicate the upper and lower ELISA detection limits, respectively. Statistical analysis was performed using Friedman test with comparison to control (0 μg/ml) by Dunn's Multiple Comparisons test. P values are represented as follows: * P<0.05, ** P<0.01.

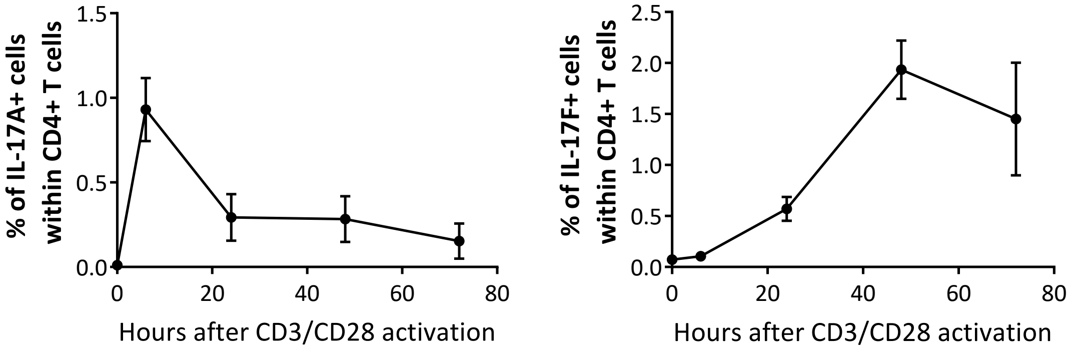

Supplementary Figure 5. Kinetics of IL-17A and IL-17F expression from CD4+ T cells. Healthy donor bulk CD4+ T cells were cultured with plate-bound anti-CD3 mAb and soluble anti-CD28 mAb for the indicated time points and then cultured with brefeldin for 3 hours. Cells were then stained for IL-17A and IL-17F expression by flow cytometry (n=3).

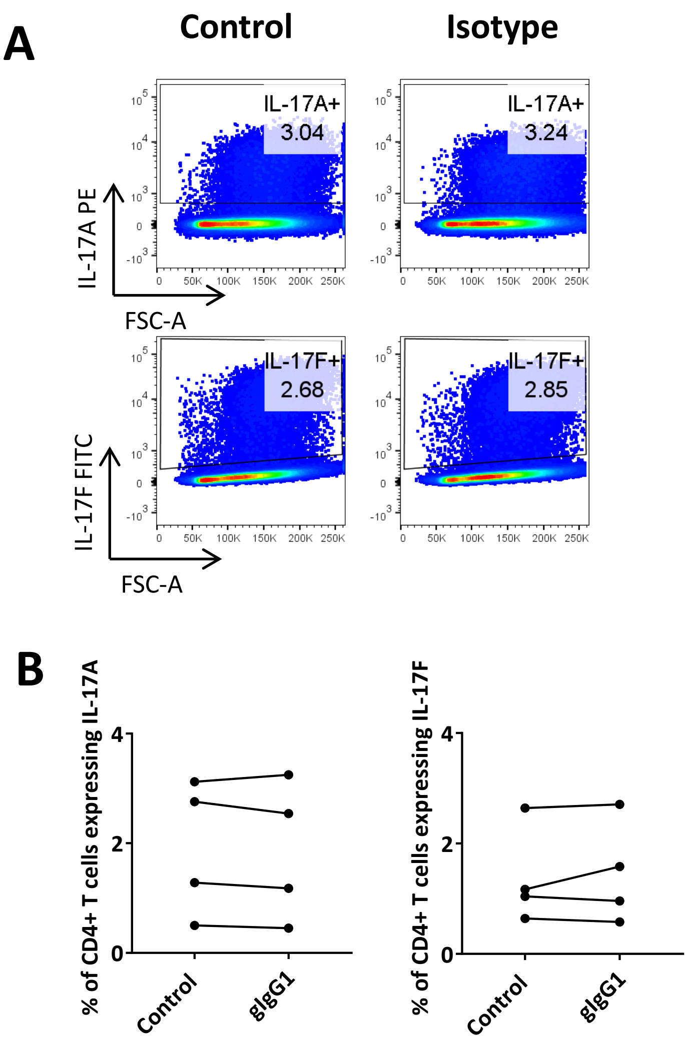

Supplementary Figure 6. Anti-IL-2 mAb isotype control (goat IgG) does not affect IL-17A+ or IL-17F+ CD4+ T cell. Bulk CD4+ T cells from healthy donors (n=4) were cultured with anti-CD3 mAb, IL-23 and IL-1β in absence (control) or presence of isotype control Ab (goat IgG1, 1 µg/ml). After 3 days cells were re-stimulated and assessed for intracellular cytokine expression. **(A)** Representative dot plots and **(B)** cumulative data show the frequencies of total IL-17A+ and IL-17F+ CD4+ T cells (n=4).

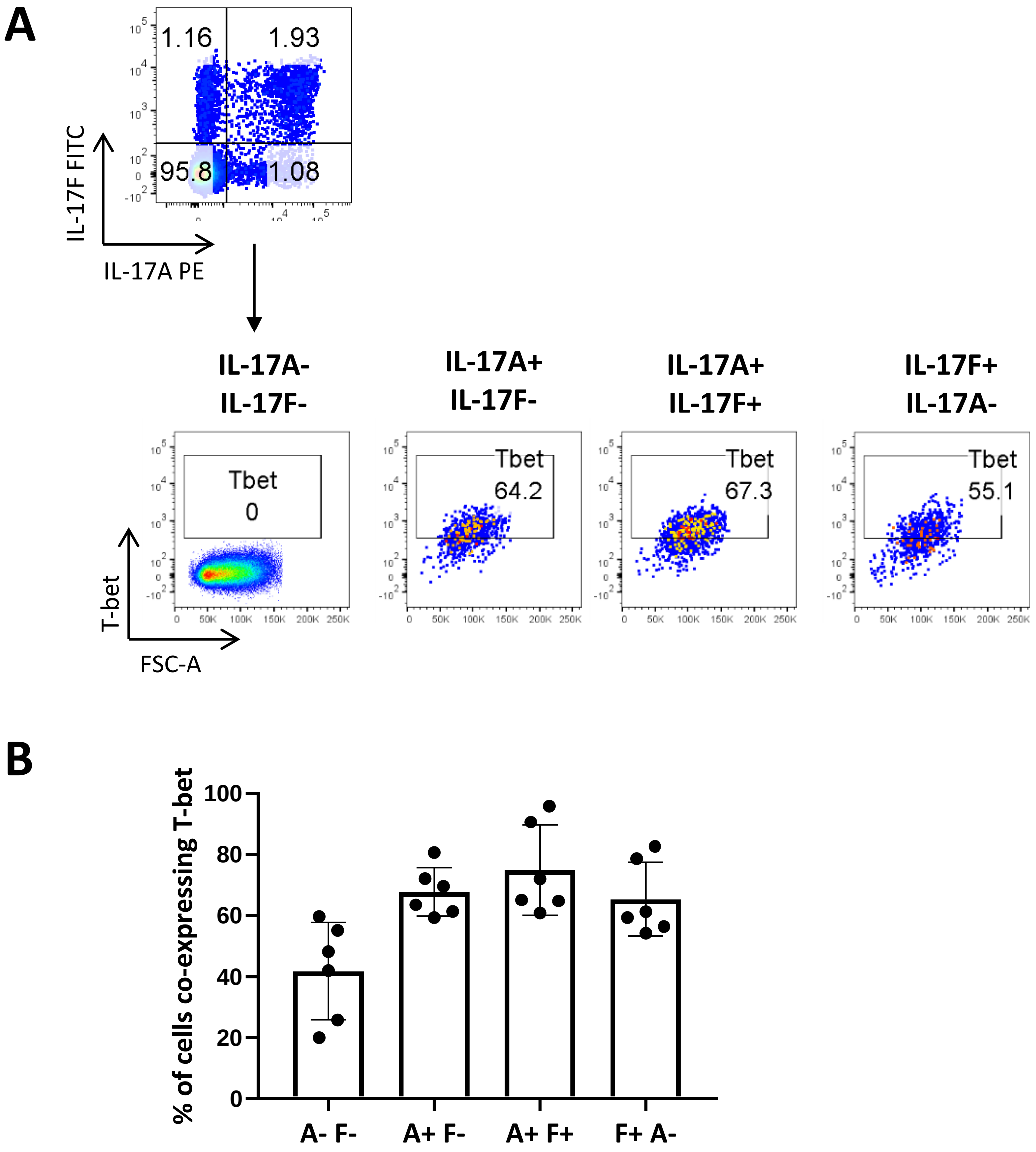

Supplementary Figure 7. Expression of T-bet in IL-17A+ and IL-17F+ CD4+ T cells. Bulk CD4+ T cells (1x10[6]) were cultured with plate-bound anti-CD3 mAb, IL-1β and IL-23. After 3 days were re-stimulated for 3 hours with PMA, ionomycin and Golgi-Stop and assessed for the expression of the transcription factor, T-bet and the expression of IL-17A and IL-17F. **(A)** Representative dot plot of IL-17A+ and IL-17F+ CD4+ T cell populations. Following gating within IL-17A-IL-17F-, IL-17A+IL-17F, IL-17A+IL-17F+ and IL-17F+IL-17A- CD4+ T cells, the expression of T-bet was analysed. **(B)** Cumulative data showing the percentage of each population expressing T-bet (n=6).

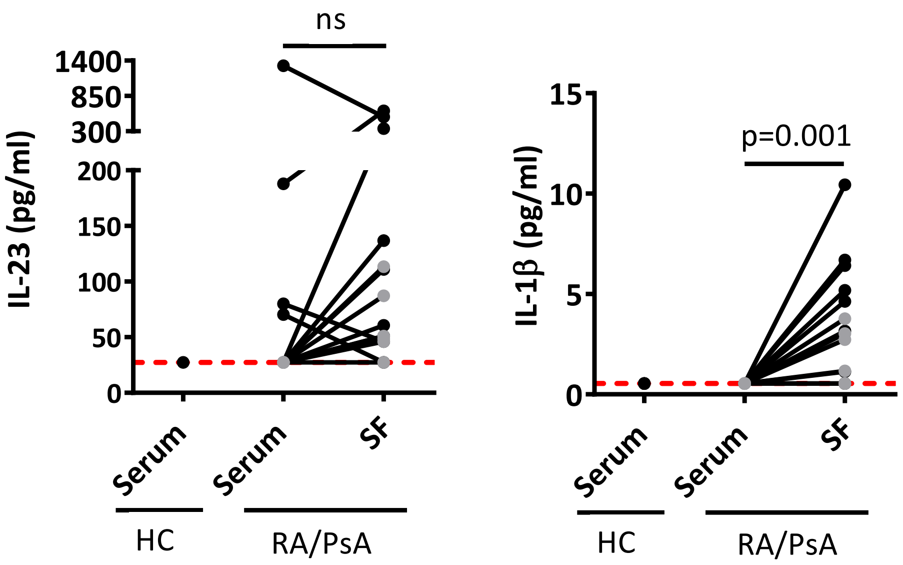

Supplementary Figure 8. Presence of IL-1β and IL-23 in inflammatory arthritis. Paired cell-free serum and synovial fluid from RA (n=7) (black symbols) and PsA (grey symbols) (n=6) patients were analysed by Luminex for the presence of IL-1β and IL-23. Dashed red lines represent the lower detection limit. Statistical significance was calculated using a Wilcoxon matched pairs test.

	RA Paired PB/SF (n = 7)	PsA Paired PB/SF (n = 6)	OA Paired PB/SF (n = 2) *	Healthy Control PB (n=2)
**Male, no.**	2	5	-	1
**Female, no.**	5	1	1	1
**Age, mean ± SEM years**	47.0 ± 0.3	36.5 ± 2.9	72	39.50 ± 8.02
**Treatment, no:**				-
No treatment	2	2	-	
MTX	1	2		
Anti-TNF	2	2	-	-
Abatacept	2	-		
**DAS28**				
Mean ± SEM	4.6 ± 0.3	4.6 ± 0.5	-	-
Range	3.5 – 5.43	1.89 – 5.78	-	-

Supplementary Table 1. Demographic and clinical parameters of patient samples included in ELISA assays.

* Gender and age not available for one OA patient.

	RA<br>Paired PB/SF<br>(n = 8)	PsA<br>Paired PB/SF<br>(n = 6)
**Male, no.**	0	4
**Female, no**.	8	2
**Age, mean ± SEM years**	54.9 ± 7.0	43.8 ± 4.2
**Treatment, no.**		
No treatment	2	1
MTX	5	3
Anti-TNF	1	1
Other (Abatacept, Ustekinumab)	-	1
**DAS28**		
Mean ± SEM	5.2 ± 0.4	4.3 ± 0.6
Range	3.8 – 7.02	1.89 – 6.0
Seronegative	2	6

Supplementary Table 2. Demographic and clinical parameters of patient samples included in flow cytometry analysis.

mAb	Conjugate	Clone	Manufacturer
CD3	PE-Cy7	UCHT1	Biolegend
CD3	FITC	SK3	Biolegend
CD4	PerCP-Cy5.5	SK3	Biolegend
CD8	Pacific Blue	RPA-T4	Biolegend
CD14	APC Vio770	TUK4	Miltenyi Biotec
IL-17A	PE	BL168	Biolegend
IL-17A	APC	eBio17B7	eBioscience
IL-17F	AlexaFluor488	Poly5166	Miltenyi Biotec
IL-17F	PE	SHLR17	eBioscience
IFNγ	Pacific Blue	4S.B3	Biolegend
IFNγ	AlexaFluor488	4S.B3	eBioscience
TNFα	APC	MAb11	Biolegend
TNFα	eFluor450	Mab11	eBioscience
IL-10	AlexaFluor647	JES3-9D7	Biolegend
GM-CSF	Pacific Blue	BVD2-21C11	Biolegend
T-bet	PE	eBIO4B10	eBioscience
T-bet	Pacific Blue	4B10	Biolegend
T-bet	PE-Cy7	eBio4B10	eBioscience

Supplementary Table 3. Flow cytometry antibodies used in this study.
